# Diagnostic performance of digital markings on head computed tomography for evaluating intracranial hypertension in neonates and infants

**DOI:** 10.1007/s11604-025-01927-x

**Published:** 2025-12-29

**Authors:** Akira Yogi, Tomohide Yoshida, Naoya Imanaga, Amiko Kayo, Erina Iha, Gyo Iida, Nanae Tsuchiya, Yukari Tomori, Akihiro Nishie

**Affiliations:** 1 Department of Radiology, Graduate School of Medical Science, University of the Ryukyus, 1076, Kiyuna, Okinawa Ginowan, 901-2725 Japan; 2 Department of Child Health and Welfare, Graduate School of Medical Science, University of the Ryukyus, Okinawa Kiyuna, Ginowan, Japan; 3 Department of Ophthalmology, Graduate School of Medical Science, University of the Ryukyus, Okinawa Kiyuna, Ginowan, Japan; 4 Department of Radiology, Naha City Hospital, Naha, Okinawa, Japan; 5 Department of Radiology, Chubu Tokushukai Hospital, Okinawa Kitanakagusuku-son, Japan

**Keywords:** Digital markings, CT digital markings, Copper beaten skull, Convolutional markings, Intracranial hypertension, ICH

## Abstract

**Purpose:**

This study aimed to investigate the diagnostic performance of digital markings appearing on head computed tomography (CT): *CT digital markings* to evaluate intracranial hypertension (ICH) in neonates and infants.

**Materials and methods:**

We retrospectively selected 270 consecutive pediatric patients (163 males and 107 females) aged 0–23 (8.0 ± 7.7) months, who underwent head CT at our institution from April 2009 to April 2023. Two board-certified observers evaluated the presence of CT digital markings, which are defined as focal depressions of inner table of the cranial bone, on CT images with reconstructed slice thickness ranging from 0.5 to 1 mm at the bone window setting. Generated volume rendering (VR) and ray-sum images were used as references. A board-certified pediatrician and ophthalmologist reviewed patients’ medical records and assessed the presence of ICH. Chi-square test was used to evaluate the relationship between CT digital markings and ICH in all patients, patients aged < 1 year, and 1 year old patients. A *p* value < 0.05 was considered as statistically significant. Sensitivity, specificity, positive predictive value (PPV), and negative predictive value (NPV) were also calculated, according to each comparison.

**Results:**

A total of 47 patients had ICH, and 128 patients demonstrated digital markings. There were no significant correlations between CT digital markings and ICH in all patients, patients aged < 1 year, or patients aged 1 year (*p* = 0.63, 0.38, and > 0.99, respectively). Sensitivity, specificity, PPV, and NPV were 0.51, 0.54, 0.19, and 0.84 for all patients; 0.51, 0.56, 0.24, and 0.81 for patients aged < 1 year old; and 0.50, 0.48, 0.08, and 0.92 for patients aged 1 year old, respectively.

**Conclusion:**

Digital markings on head CT observed in neonates and infants are not associated with ICH and thus not clinically useful for the diagnosis of ICH.

**Supplementary Information:**

The online version contains supplementary material available at 10.1007/s11604-025-01927-x.

## Introduction

Intracranial hypertension (ICH) is defined as elevated pressure within the cranium [1] and is classified into primary (idiopathic) and secondary ICH, depending on the etiology of the increased intracranial pressure [1]. With secondary ICH, the elevated intracranial pressure can be rapidly resolved once the underlying conditions causing these conditions are resolved.

*Digital markings*, also known as *copper beaten skull*, are imaging findings of *skull radiographs* and supposed to associate with ICH [2]. Gyral impressions on the inner table of the skull cause digital markings. However, digital markings also occur in normal pediatric patients aged between two and 14 years old, and are also known as convolutional markings [3]. The brain grows more rapidly than skull, with the brain volume approaching that of skull, resulting in the appearance of digital markings [4–6]. Copper beaten skulls refer to prominent convolutional markings; however, it is hard to differentiate copper beaten skulls and digital markings on skull radiograph. Therefore, ICH diagnosis should be made based on a comprehensive consideration of not only imaging findings but also patient’s symptoms and ophthalmic examination results [7]. In addition, digital markings are rare in normal pediatric patients younger than 2 years old [2, 3].

Head computed tomography (CT) is currently more commonly performed compared to skull radiograph as the initial diagnostic imaging modality for the examination of patients with suspected ICH. This is because CT enables the detection of the underlying CNS disorder, including cerebral hemorrhage, brain tumor, hydrocephalus, and craniosynostosis. Multiple focal depressions of the inner table of the cranial bone, which we termed “*CT digital markings”*, are routinely observed on CT in clinical practice [8–10]. CT digital markings are sometimes considered suggestive of ICH, especially in patients younger than 2 years old, due to the knowledge of digital markings on skull radiograph. However, to the best of our knowledge, the frequency and the association between CT digital markings and ICH have never been reported. We hypothesized that CT digital markings occur in normal neonates and infants younger than 2 years old and that there might be no significant correlation between CT digital markings and ICH. To assess these hypotheses, we aimed to investigate the diagnostic performance of CT digital markings for the evaluation of ICH in neonates and infants.

## Materials and methods

### Institutional review board (IRB) approval and informed consent

This research was conducted in compliance with ethical standards and approved by the IRB of our institute. The need for written informed consent and signed Patient Consent-to-Disclose Form was waived because the exams were deemed clinically relevant to patient care. Patients and their families were provided with the opportunity to opt out of study.

### Patients

We retrospectively included patients younger than 2 years old, who underwent head CT and subsequently had a medical examination by pediatricians or ophthalmologists, for clinical purposes from April 2009 and April 2023. Patients without follow-up medical examination records or available clinical information were not selected. If the patients underwent several CT examinations, the first one was used for the analysis.

A board-certified pediatrician, who was blinded to the imaging findings, reviewed the medical records to evaluate whether the patients demonstrated the ICH symptoms, including headache, blurred vision, vomiting, neck and shoulder pain, feeding difficulty, double vision, loss of vision, or blind spots. Clinical findings which indicate ICH, including bulging fontanelle, increased head circumference, suture separation, and sunset sign, were also reviewed. Some patients underwent ophthalmological examination to evaluate the presence of papilledema, which is one of the important clinical findings for ICH, by the attending pediatrician. For such patients, a board-certified ophthalmologist, who was also blind to the imaging findings, reviewed the medical records including ophthalmoscopy to determine whether papilledema was present [11]. If the patients underwent subsequent management of diuretics and/or acetazolamide administration and the symptoms were improved, we determined the patient had ICH.

### CT examinations

CT scans were performed using a 64-detector row CT (Lightspeed VCT, GE Healthcare, Milwaukee, Wisconsin, U.S.A.), a 320-detector-row CT (Aquilion ONE, Canon Medical Systems, Otawara, Japan), a 180-detector row CT (Aquilion Prime, Canon Medical Systems, Otawara, Japan), and a 160-detector row CT (Aquilion Precision, Canon Medical Systems, Otawara, Japan). The detector element was 0.5 × 0.5 mm at the isocenter. Tube voltage and tube current were 120 kV and 270 mA. Reconstructed slice thickness was from 0.5 to 1 mm in each CT scanner. Detailed scan parameters are shown in Supplemental 1.

### Visual evaluation

**Fig. 1:**
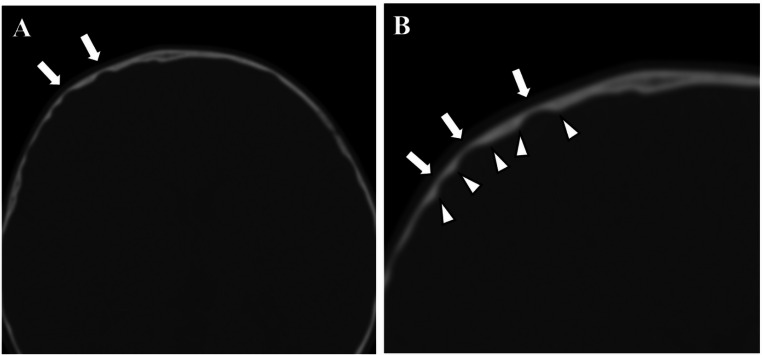
Imaging appearance of CT digital markings. **A** There are some focal depressions of the inner table in the right side of frontal bone, compared to the contralateral side, defined as CT digital markings (arrows). **B** Magnified image of right side of frontal bone demonstrates that CT digital markings well demarcated concavity. Note that the concavities exhibit relatively steep peripheral margins (arrowheads) and a smoothly contoured base, where the depth of the focally depressed area is thinner than half of that at the adjacent normal skull bone (arrows)

Two board certified radiologists independently reviewed the patients’ axial head CT images with bone window settings and evaluated the presence of CT digital markings. Subsequently, they evaluated CT digital markings again, in consensus. We defined the CT digital markings as one or more well demarcated concavity involving the inner table of the cranial bone, exhibiting relatively steep peripheral margins and a smoothly contoured base, where the depth of the focally depressed area is thinner than half of that at the adjacent normal skull bone (Fig. 1). Locations of CT digital markings were also recorded, according to cranial bones: frontal, parietal, occipital, and temporal bones, using both volume rendering (VR) and ray-sum images generated by VINCENT ver. 6.7 (FUJIFILM Medical Japan, Tokyo, Japan) as reference for determining the location of CT digital markings. We kept the default settings for generating these reconstructed images by just clicking the standard buttons in the VINCENT viewer without further adjustment, constant to avoid evaluation bias. If there was a discrepancy between the axial images and reconstructed images, according to the presence of CT digital markings, we applied the results from axial images. The number of affected cranial bones were counted.

The presence of intracranial tumors, hemorrhage, hydrocephalus, or craniosynostosis, which may cause ICH, was also evaluated. The presence of other central nervous system (CNS) disorders was also recorded.

### Statistical evaluation

Patients were categorized into three groups, according to the age at CT examinations: all patients, patients < 1 year old, and 1 year old patients, because head circumference rapidly grows until 1 year old, and its growth rate decrease gradually [12, 13]. Interclass correlation coefficient (ICC) was used to assess the interobserver agreement. Fisher’s exact test was used to evaluate the relationship between ICH and CT digital markings. The number of cranial bones affected by CT digital markings were compared between patients with and without ICH, using Mann-Whitney test. In addition, the correlation between the number of affected cranial bones and the presence of ICH was evaluated, using Chi-square test. These statistical analyses were performed in all groups. A *p* value < 0.05 was considered as statistically significant. Sensitivity, specificity, positive predictive value (PPV), and negative predictive value (NPV) were also calculated, according to each comparison.

## Results

**Fig. 2:**
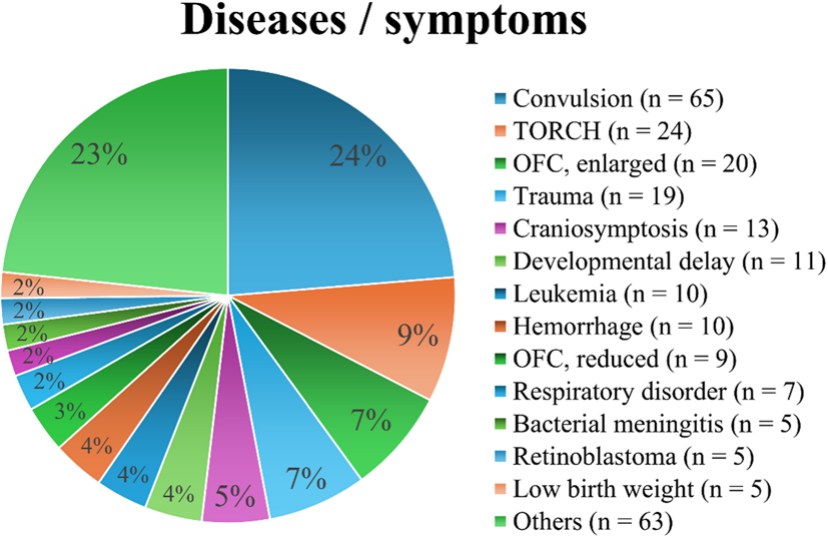
Diseases or symptoms for head computed tomography examinations. “Others” consists with brain tumor (*n* = 3), late closure of the anterior fontanel (*n* = 3), facial nerve palsy (*n* = 3), agenesis of corpus callosum (*n* = 3), congenital skull malformation (*n* = 3), Chiari malformation (*n* = 3), osteogenesis inperfecta (*n* = 3), hypoxic encephalopathy (*n* = 3), congenital hearing loss (*n* = 2), meningocele (*n* = 2), skull deformity (*n* = 2), propionic acidemia (*n* = 2), 21 trisomy (*n* = 2), Sturge-Weber syndrome (*n* = 2), disturbance of consciousness (*n* = 2), tuberous sclerosis complex (*n* = 2), achondroplasia (*n* = 2), neonatal asphyxia (*n* = 2), leukocoria (*n* = 2), suckling difficulty (*n* = 1), glio-ependymal cyst (*n* = 1), meatal atresia (*n* = 1), arachnoid cyst (*n* = 1), cranial bone tumor (*n* = 1), mitochondrial disease (*n* = 1), glaucoma (*n* = 1), Horner’s syndrome (*n* = 1), screening for metastatic tumor (*n* = 1), vomiting (*n* = 1), cataract (*n* = 1), uncertain encephalitis (*n* = 1), hemiplegia (*n* = 1), congenital cystic adenomatoid malformation of the lung (*n* = 1), Pierre-Robin syndrome (*n* = 1), Krabbe disease (*n* = 1), and cerebral infarction (*n* = 1). Abbreviations: CMV = cytomegalovirus, OFC = occipito-frontal circumference, TORCH = toxoplasmosis, other, rubella, cytomegalovirus, and herpes simplex

A total of 1,050 head CT examinations were performed on 699 patients younger than 2 years old, during the study period. A total of 429 patients with 578 CT scans were excluded from the selection, because they were ordered by other department or were not evaluated for the presence of ICH by pediatricians or ophthalmologists. As a result, 270 patients were selected for the analysis, including 163 males and 107 females aged 0 to 23 months (8.0 ± 7.7). The most common disease or symptoms for the CT examinations were convulsions (*n* = 65), followed by toxoplasmosis, others (syphilis, Hepatitis B), rubella, cytomegalovirus, and herpes simplex (TORCH) (*n* = 24); enlarged occipito-frontal circumference (*n* = 20), head trauma (*n* = 19), and craniosynostosis (*n* = 13) (Fig. 2).

Additionally, 209, 57, 3, and 1 patients underwent CT examinations by Lightspeed VCT and Aquilion ONE, Prime, and Precision, respectively. The minimum, maximum, and mean value of CTDI_vol_ and DLP ranged from 2.0 to 78.0 (mean, 34.5); and 38.9 and 471.4 (mean, 275.8). Furthermore, 16 patients had intracranial hemorrhages, 15 had hydrocephalus, 6 had craniosynostosis, and 5 had intracranial tumors.

ICH was diagnosed with 47 patients (17.4%). The ICH diagnosis was made by clinical symptoms and examinations in all patients. In one patient, we found that the intracranial pressure measurement was performed but could not find the measured ICP value.

In the evaluation for CT digital markings by two observers, the ICC value 0.69, indicating moderate to good agreement. According to the evaluation of each cranial bone, the ICCs were 0.71 for the parietal bone, 0.84 for the frontal bone, 0.59 for the occipital bone, and 0.49 for the temporal bone. Subsequent consensus evaluation revealed that CT digital markings on head CT were identified in 127 patients, including 24 patients with ICH (Table 1A). In the patients not greater than 1 year old group, ICH was diagnosed with 41 patients. CT digital markings were observed in 88 patients, including 21 patients with ICH (Table 1B). In the patients aged 1 year old group, ICH was diagnosed with 6 patients. CT digital markings were observed in 39, including 3 patients with ICH (Table 1C). The occipital, parietal, frontal, and temporal bones were affected in 77, 72, 43, and 15 patients in all patients group; in 48, 59, 43, and 7 patients in patients not greater than 1 year old group; and in 29, 13, 18, and 5 patients in patients aged at 1 year old group, respectively. The numbers of affected cranial bones were 1, 2, 3, or 4 in 60, 55, 11 and 1 patients in all patients group; in 42, 39, 6, and 1 in patients not greater than 1 year old group; in 18, 16, 5, and 0 patients in patients aged at 1 year old group. Specifically, the numbers of affected cranial bones in patients with ICH were 1, 2, 3, or 4 in 7, 13, 4, and 0 in all patients group; in 6, 12, 3, or 0 in patients not greater than 1 year old group; in 1, 1, 1, and 0 patients in patients aged at 1 year old group (Table 2).

**Table 1:** Association between intracranial hypertension and CT digital markings

			ICH		
			Present		Not present
(A) All patients					
CT digital markings	Present		24		103
	Not present		23		120

(B) Patients not greater than 1 year old					
CT digital markings	Present		21		67
	Not present		20		120

(C) Patients aged 1 year old					
CT digital markings	Present		3		36
	Not present		3		33

**Table 2:** Number of affected cranial bones with CT digital markings

		ICH				
		Present			Not present	
(A) All patients						
No. of affected cranial bones	0	23			120	
	1	7			53	
	2	13			42	
	3	4			7	
	4	0			1	

(B) Patients not greater than 1 year old						
No. of affected cranial bones	0	20			87	
	1	6			36	
	2	12			27	
	3	3			3	
	4	0			1	

(C) Patients aged 1 year old						
No. of affected cranial bones	0	3			33	
	1	1			17	
	2	1			15	
	3	1			4	
	4	0			0	

**Fig. 3:**
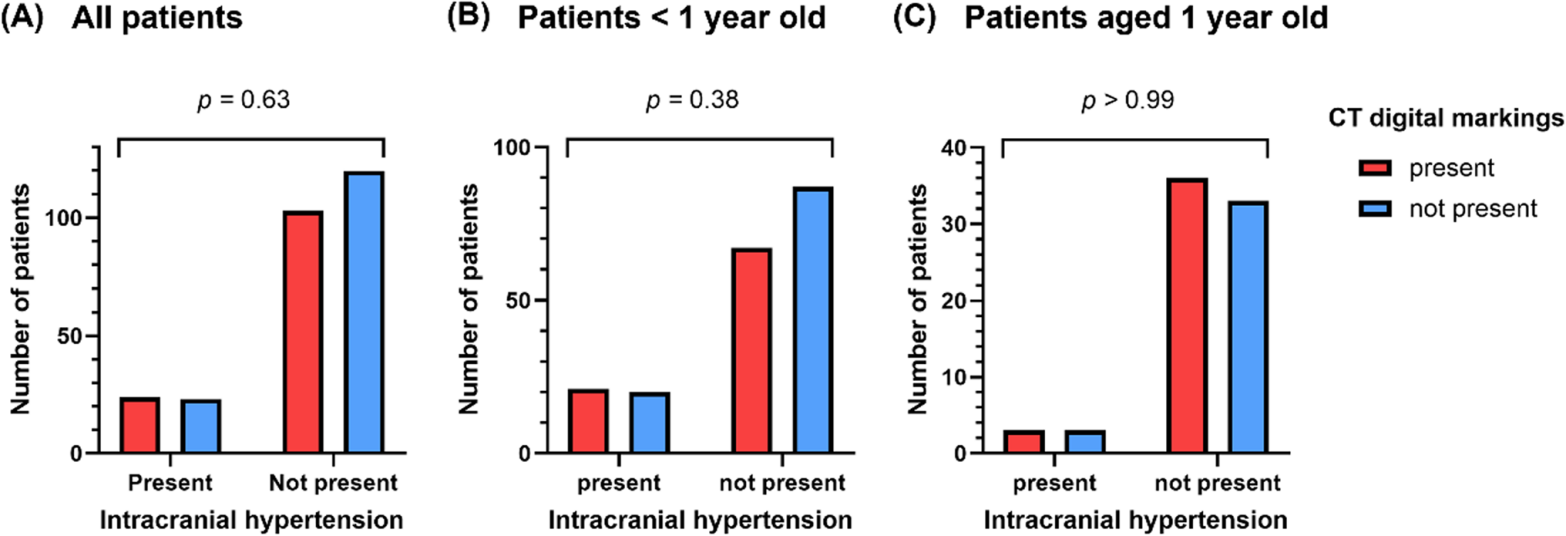
Box plot and whisker graphs demonstrating the relationship between intracranial hypertension and CT digital markings. There were no significant correlations between intracranial hypertension and CT digital markings in (**A**) all patients, (**B**) patients not greater than one year old, and (**C**) patients aged at one year old

**Fig. 4:**
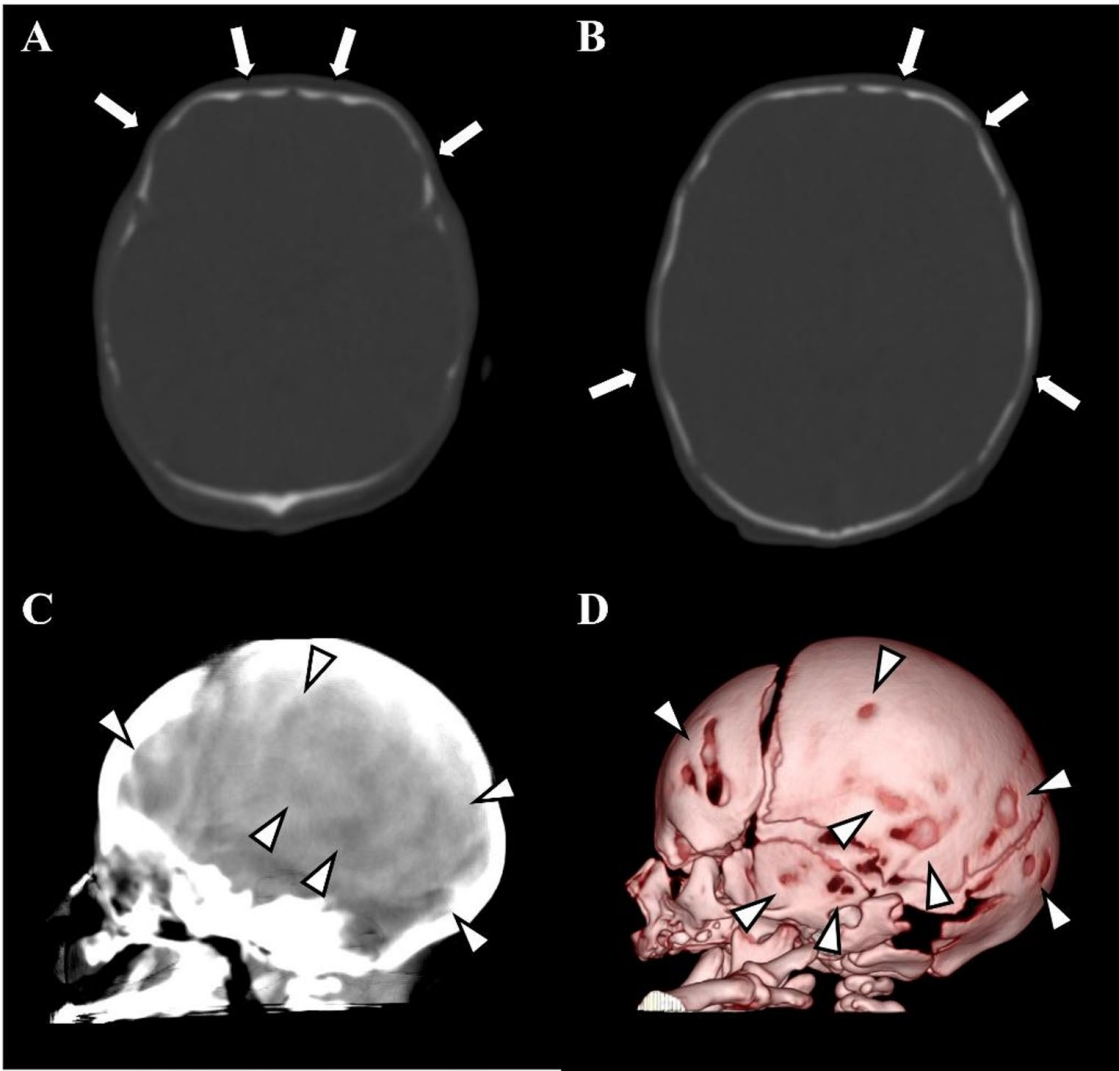
A zero-day old girl who demonstrated intracranial hypertension and was suspected of having intracranial hemorrhage

**Fig. 5:**
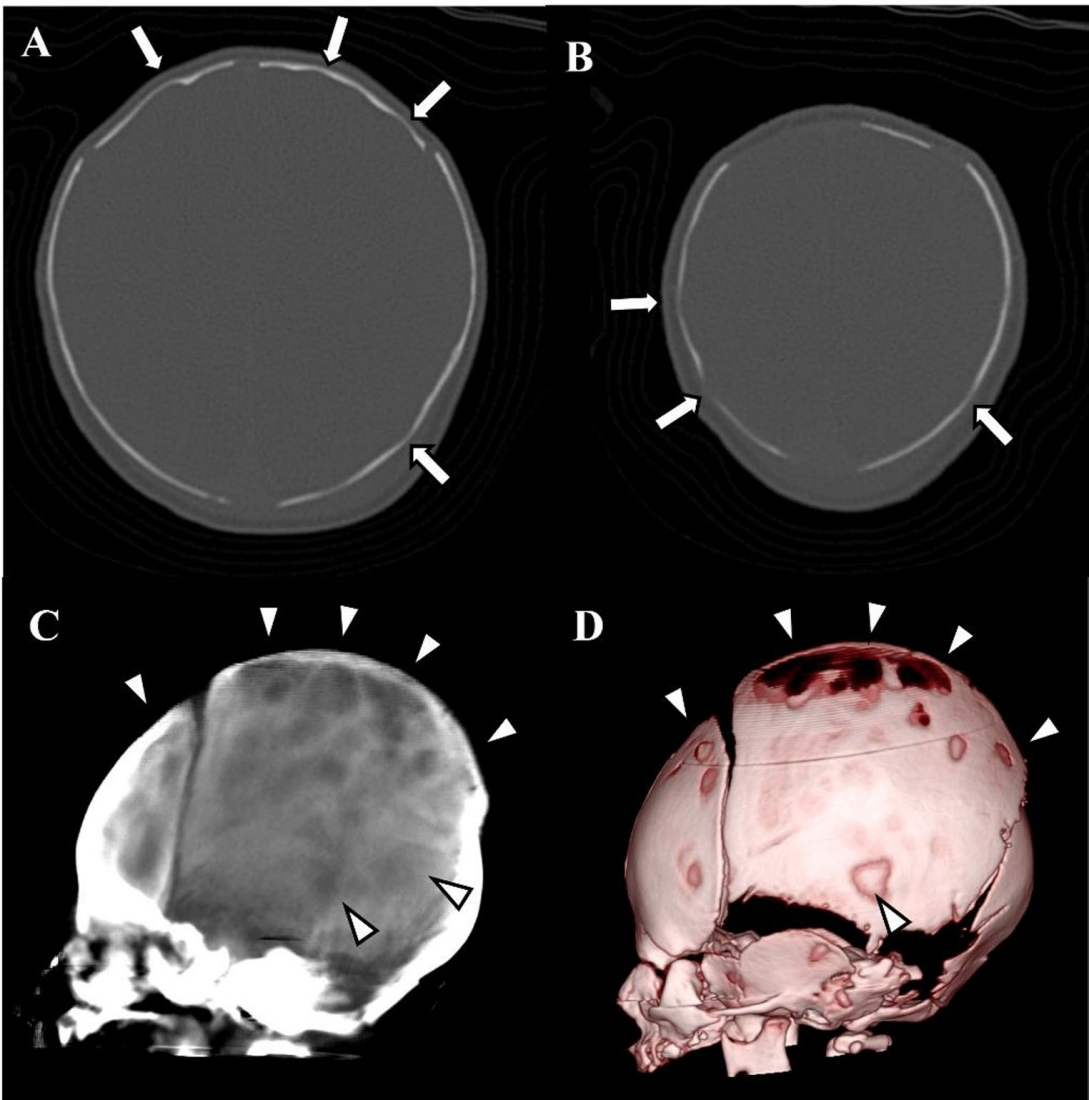
A zero-day-old boy with head trauma, showing no traumatic changes on CT or ICH symptoms

**Fig. 6:**
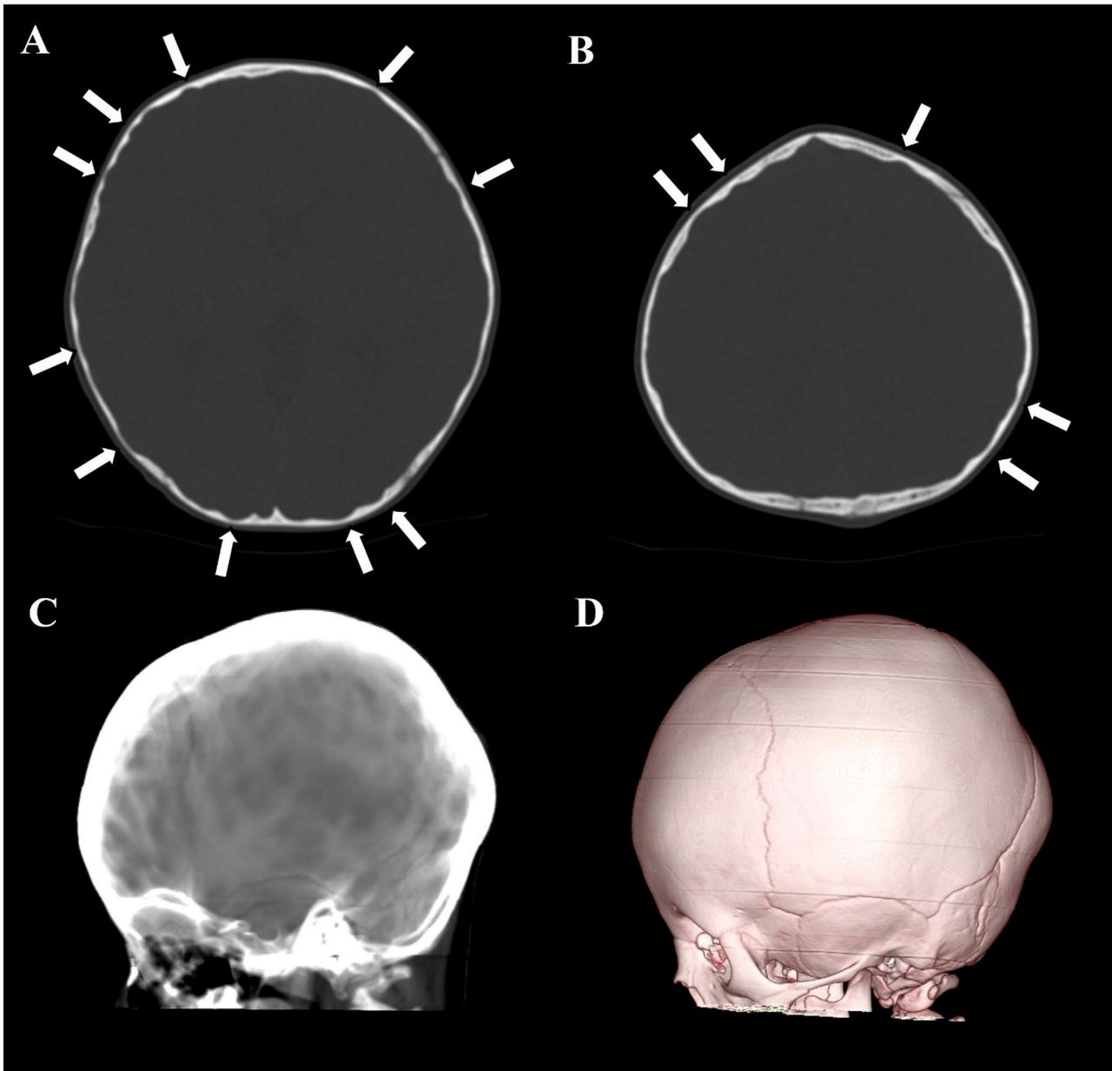
A 23-month-old boy with craniosynostosis demonstrating ICH. **A**, **B** Axial head CT images with bone window setting demonstrating multiple CT digital markings whole skull vault (arrows). **C** Ray-sum image demonstrating multiple focal hyperlucent areas due to CT digital markings entirely. **D** VR image, however, demonstrates no decompressions or holes, suggesting CT digital markings. Note that all cranial sutures are fused by craniosynostosis

**Fig. 7:**
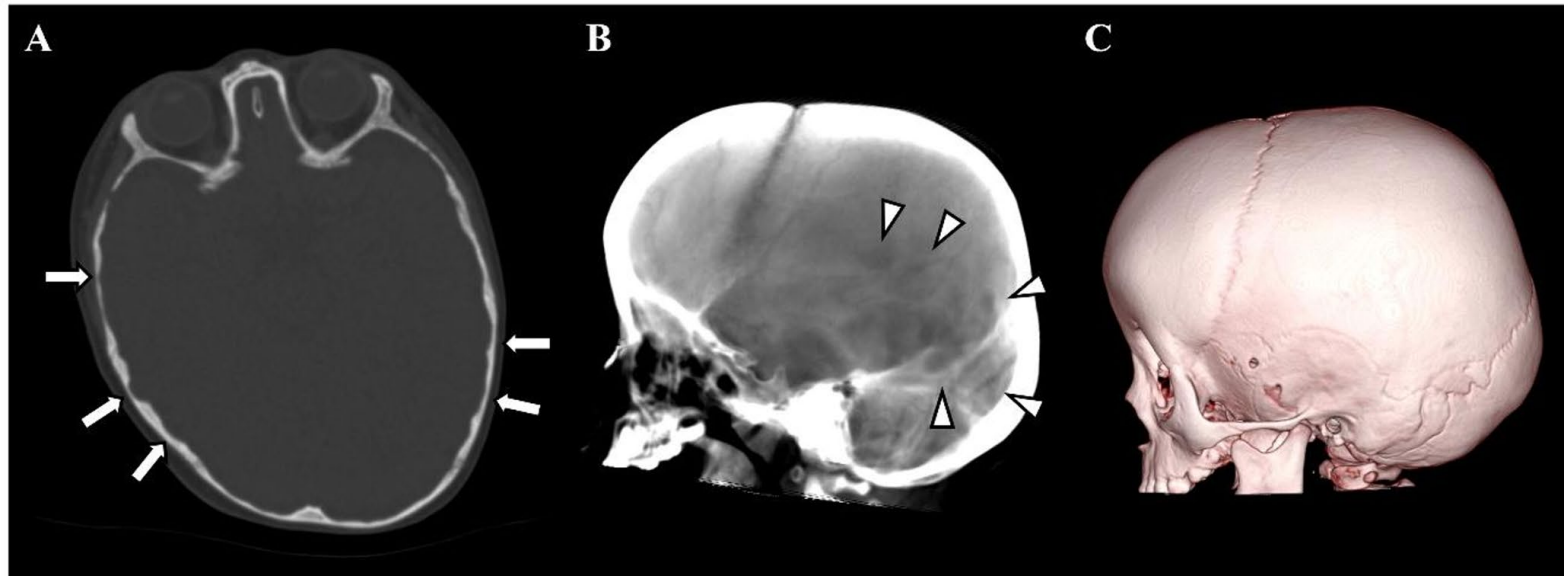
A 22-month-old boy with occipito-frontal circumference, enlarged, who did not show ICH. **A** Axial head CT image demonstrating multiple CT digital markings at bilateral temporal and parietal bones (arrows). **B** Ray-sum image demonstrates multiple focal hyperlucent areas due to CT digital markings at posterior side of parietal bone and occipital bone (arrowheads). **C** VR image demonstrating no focal decompression or holes. This may have been due to the external table of cranial bone growing and being thick enough to be recognized by the image reconstruction software: VINCENT

There were no significant correlations between CT digital markings and ICH (all patients: *p* = 0.63, patients not greater than 1 year old: *p* = 0.38, patients aged at 1 year old: *p* > 0.99) (Figs. 3, 4, 5, 6 and 7). The number of cranial bones affected by CT digital markings were not significantly different between with and without ICH (all patients: *p =* 0.21, patients not greater than 1 year old: *p* = 0.14, patients aged at 1 year old: *p* = 0.88). There were no correlations between the number of affected cranial bones and ICH in all patients (*p* = 0.20), patients not greater than 1 year old (*p* = 0.14), and patients aged at 1 year old (*p* = 0.75).

All age groups demonstrated relatively high NPV: 0.84, 0.81, and 0.92 in all patients, patients not greater than one year old, and patients aged one year old, respectively. In contrast, sensitivity, specificity, and PPV were low in all groups (Table 3).

**Table 3:** Diagnostic performance of the CT digital markings for intracranial hypertension

Performance	All patients	Patients not greater than 1 year old	Patients aged 1 year old
Sensitivity	0.51	0.51	0.50
Specificity	0.54	0.56	0.48
PPV	0.19	0.24	0. 08
NPV	0.84	0.81	0.92

## Discussion

We demonstrated that CT digital markings occur in patients younger than 2 years old with or without ICH. Furthermore, there was no significant relationship between CT digital markings and ICH. The number of affected bones showed no significant difference between with and without ICH groups and also showed no significant correlation with ICH. Additionally, the diagnostic performance of digital marking for ICH was limited, demonstrating low sensitivity, specificity, and PPV, even in the presence of relatively higher NPV.

Schreiber reported that digital markings observed on a *skull radiograph* correlated with papilledema and hypothesized that this imaging finding was associated with chronically elevated intracranial pressure [14]. However, the exact correlation between digital markings on skull radiographs and ICH was not demonstrated. Davidoff and Gass reviewed over 2,500 skull radiographs and found that the digital markings occur in neurologically normal pediatric patients [4]. Digital markings are not observed in patients < 8 months old and have a reduced incidence in patients > 18 months old. Notably, the incidence of digital markings rapidly increased until 4 years of age, maintained between the ages of 7–9 years, and rapidly decreased to reach the lowest incidence in 14 year old patients [4]. Macaulay reported that the incidence of digital markings correlated with brain weight [3]. He also evaluated 440 skull radiographs of neurologically normal pediatric patients to rate the digital markings using a three-point visual scale and reported that the incidence of prominent digital markings increased until 6 years of age and decreased after 7 years of age, while those with few or no markings increased. Macaulay also reported that the percentage of little or no digital markings was over 60% and 20% in first and second year of life, respectively, indicating that digital markings could occur in patients under 2 years old [3]. Therefore, the skull radiograph finding of digital markings is not a useful tool for the diagnosis of ICH.

In this study, we applied CT, for the first time, to evaluate the diagnostic performance of digital markings on CT. CT is a more useful tool than radiographs for the evaluation of normal structures and pathological lesions, including skull [15]. It was thus hypothesized that CT could help better visualize the subtle morphological changes in inner table of the skull (including CT digital markings), which may be missed on skull radiographs of infants and neonates.

Tuite reviewed skull radiograph of 123 pediatric patients with craniosynostosis and explored the relationship between the extent of digital markings and ICH. They found that a diffuse digital markings was seen more commonly in patient with craniosynostosis compared to age- and gender-matched controls, though the presence of digital marking was no more common in patients with craniosynostosis [2]. They evaluated the extent of digital markings whether these findings were present at greater than or not greater than 50% of anterior or posterior side of cranium. In our study, we counted the number of cranial bones affected by CT digital markings to evaluate the extent, instead of evaluating the rate of affected area in cranium ( ≦ 50%, < 50%), because it was hard to define the rate of affected area by percentages on CT. Subsequently, we found that there was no significant difference in number of affected bones between patients with and without ICH or no significant correlation between the number of affected bones and the presence of ICH. It is also supposed to result from better visualizing performance of CT. Focal compressions of inner table of the skull derived from normal development of brain and ICH were visualized more than skull radiograph.

CT digital markings did not correlate with ICH in infants and neonates or older pediatric patients. The etiology of CT digital markings in infants and neonates is unknown. One possible hypothesis is that undeveloped membranous ossification of the skull vault in neonates and infants may cause the depression of inner table, which is similar to the dysplasia of the membranous skull vault in *lacunar skull*, which is commonly seen with Chiari II malformation [6, 16]. Lacunar skull could appear as well-defined radiolucent areas in the skull vault at birth and spontaneously disappear by 6 months of age [6, 16]. It is difficult to differentiate lacunar skull and digital markings on skull radiograph, therefore, clinical information on age and Chiari II malformation enables us to differentiate these two statuses. However, there is also no study evaluating the lacunar skull on CT, meaning that there could be focal decompression of inner table on CT, due to lacunar skull, in patients over the age of six months. In conclusion, CT digital markings are not useful biomarkers for ICH. If the head CT of patients younger than 2 years old without any ICH symptoms or CNS disorders, which could cause ICH including craniosynostosis, hydrocephalus, hemorrhage, and tumors, demonstrated CT digital markings, it is not clinically appropriate or useful to predict the possible ICH.

As stated above, NPV of CT digital markings for predicting ICH was relatively high (0.81–0.92), though sensitivity, specificity and PPV were quite low. It indicates that we do not have to care about ICH, if there were no CT digital markings in neonates and infants. However, we should be aware that NPV of a diagnostic examination is significantly affected by the prevalence of the condition, where NPV for the condition with lower frequency tends to be higher [17]. The incidence of ICH was 17.4%, which was relatively lower, in spite of the common pathological status. Relatively high NPV could be influenced by such low incidence and may not be able to apply to other cohort directly.

There are some other imaging findings to correlate with intracranial pressure [18–23]. Optic nerve sheath is continuous to the meninges of the cranium and is encased with the subarachnoid membrane, so that cerebrospinal fluid (CSF) can move into the subarachnoid space in the optic nerve sheath; therefore, the optic nerve sheath diameter (ONSD) increases in ICH patients [18–20]. Although the usefulness of ONSD in adult subject is reported, there is limited evidence in children, especially in neonates and infants [20, 21]. It has been reported that the patency of basal cistern on CT is also useful to predict ICH in pediatric patients with traumatic brain injury (TBI) [22, 23]. However, Kouvarellis AJ et al. reported that basal cistern could be prominent even in patients with severe TBI [23]. We should be careful to use these findings to predict ICH, as well as CT digital markings; however, combining these findings may strengthen the performance of predicting ICH. Further analyses with more patients are warranted.

This study has some limitations. First, there is no definite and established definition for CT digital markings since this is the first study focusing on the correlation between CT digital markings and ICH. We defined the markings as focal depressions in the inner table of the cranial bone, where the thickness is less than half that of the adjacent cranial bone, which is simple and useful for visual evaluation. Even though deeper or more shallow depression might correlate with ICH, it is hard to define and is thus not clinically useful. Second, other imaging findings for diagnosing ICH, enlargement of anterior fontanel and sella turcica demineralization of tuberculum, were not evaluated. These findings have never been evaluated the correlation with ICH on head CT as well. Independent evaluation for each finding and comprehensive evaluation using all findings may help evaluate their association with ICH. Third, the number of digital markings was not evaluated as it was challenging and would not help the management of neonates and infants in a clinical practice. Fourth, only patients younger than 2 years old were analyzed as this is the age group previously associated with a correlation between digital markings and ICH. However, it is essential to evaluate older pediatric patients (> 2 years old) to analyze the nature of CT digital markings. As we confirmed that CT digital markings occur normally in neonates and infants, the incidence of this imaging finding in older patients could be different from that in skull radiograph: digital markings increase up to 6 years old; and after the age of seven the number with prominent markings decreases [3]. Further analysis with more patients is needed. Fifth, we did not evaluate the relationship between CNS disorders, including intracranial tumors, hemorrhage, or hydrocephalus, and the presence of CT digital markings, because the numbers of patients with each disease were low. Further analysis with more patients with such disease is also needed. Sixth, the pediatrician reviewing the medical records in this study could access physician’s notes based on the CT reports generated by radiologists, which could be a potential confounding factor. Lastly, this was a retrospective study and has the inherent limitations associated with this study method.

## Conclusion

The presence of CT digital markings, which are focal depressions of inner table of the cranial bone, appears normally in neonates and infants. Furthermore, it does not significantly correlate with ICH, as well as digital markings on skull radiograph do not in patients over two years old. It is thus not clinically useful to assess CT digital markings for the evaluation of ICH and should be avoided not to mislead the management of patients with neonate and infants. 

## Supplementary Information

Below is the link to the electronic supplementary material.

Supplementary Material 1

## Data Availability

The data that support the findings of this study are not openly available due to reasons of sensitivity and are available from the corresponding author upon reasonable request. Data are located in controlled access data storage at University of the Ryukyus Hospital.
