# Supplementary material for: Diagnostic performance of digital markings on head computed tomography for evaluating intracranial hypertension in neonates and infants
Source: Jpn J Radiol. 2025 Dec 29;44(5):895–904. doi: 10.1007/s11604-025-01927-x (PMC13144279; doi:10.1007/s11604-025-01927-x)
Supplement: Supplementary file 1 — Supplementary Material 1 [file 11604_2025_1927_MOESM1_ESM.docx]

**Supplemental. CT scan parameters**

**Lightspeed VCT**

64 detector rows

Detector element: 0.5 × 0.5 mm at the isocenter

Small focal spot of the X-ray tube was 0.4 mm x 0.5 mm

Slice thickness 0.5 × 64 row non-helical scan

Tube voltage 120 kv

Tube current 270 mA

Tube rotation time 0.4 seconds, and 512 × 512 reconstructed image matrix.

**Aquilion ONE**

320 detector rows

Detector element: 0.5 × 0.5 mm at the isocenter

Small focal spot of the X-ray tube was 0.9 x 0.8 mm

Slice thickness 0.5 × 80 row non-helical scan

Tube voltage 120 kv

Tube current 270 mA

Tube rotation time 0.5 seconds, and 512 × 512 reconstructed image matrix.

**Aquilion Precision**

160 detector rows

The detector matrix: 896 channels × 80 rows

Detector element: 0.5 × 0.5 mm at the isocenter

Small focal spot of the X-ray tube was 1.6 x 1.4 mm

Slice thickness 0.5 × 80 row non-helical scan

Tube current 270 mA

Tube voltage 120 kv

Tube rotation time 0.5 seconds, and 512 × 512 reconstructed image matrix.

**Aquilion Prime**

80 detector rows

The detector matrix: 896 channels × 80 rows

Detector element: 0.5 × 0.5 mm at the isocenter

Small focal spot of the X-ray tube was 0.9 x 0.8 mm

Slice thickness 0.5 × 80 row non-helical scan

Tube voltage 120 kv

Tube current 270 mA

Tube rotation time 0.5 seconds, and 512 × 512 reconstructed image matrix.
